# Supplementary material for: The Gut Bacteria Dysbiosis Contributes to Chronic Graft-Versus-Host Disease Associated With a Treg/Th1 Ratio Imbalance
Source: Front Microbiol. 2022 Sep 8;13:813576. doi: 10.3389/fmicb.2022.813576 (PMC9493085; doi:10.3389/fmicb.2022.813576)
Supplement: Supplementary file 1 [file Data_Sheet_1.zip › P101SC18090073-01-B1-3-4_result/05.WebShow/taxa_charts_html/charts/08fADyj31QPMsTw9skjKMZ75wxZz8K_legend.pdf]

k__Bacteria;Other;Other;Other
k__Bacteria;p__Actinobacteria;c__Rubrobacteria;o__Rubrobacterales
k__Bacteria;p__Bacteroidetes;c__Bacteroidia;o__Bacteroidales
k__Bacteria;p__Firmicutes;Other;Other
k__Bacteria;p__Firmicutes;c__Bacilli;o__Lactobacillales
k__Bacteria;p__Firmicutes;c__Clostridia;o__Clostridiales
k__Bacteria;p__Firmicutes;c__Erysipelotrichia;o__Erysipelotrichales
k__Bacteria;p__Firmicutes;c__Negativicutes;o__Selenomonadales
k__Bacteria;p__Fusobacteria;c__Fusobacteriia;o__Fusobacteriales
k__Bacteria;p__Oxyphotobacteria;c__unidentified_Oxyphotobacteria;o__unidentified_Oxyphotobacteria
k__Bacteria;p__Proteobacteria;c__Alphaproteobacteria;o__unidentified_Alphaproteobacteria
k__Bacteria;p__Proteobacteria;c__Deltaproteobacteria;o__Desulfovibrionales
k__Bacteria;p__Proteobacteria;c__Gammaproteobacteria;o__Enterobacteriales
k__Bacteria;p__Proteobacteria;c__Gammaproteobacteria;o__Pasteurellales
k__Bacteria;p__Proteobacteria;c__Gammaproteobacteria;o__Pseudomonadales
k__Bacteria;p__Proteobacteria;c__Gammaproteobacteria;o__unidentified_Gammaproteobacteria
k__Bacteria;p__Tenericutes;c__Mollicutes;o__
k__Bacteria;p__Verrucomicrobia;c__Verrucomicrobiae;o__Verrucomicrobiales
k__Bacteria;p__unidentified_Bacteria;c__unidentified_Bacteria;o__Campylobacterales
